# Is Dentistry a Financial Success?

**Published:** 1892-04

**Authors:** 


### Editorial.

### IS DENTISTRY A FINANCIAL SUCCESS?

The inquiry, Is dentistry a paying profession? seems to be agitating some of our dental societies; and, as faithful exponents of the current thought, it is our duty to meet this question, not, perhaps, that additional light may be thrown upon it, but that it may receive the general consideration the importance of the subject demands.

The rapid increase in the number of young men entering dentistry has naturally led to the pessimistic feeling that the flood is upon us, and we are doomed to an overflow, not only of incompetency, but a bitter competition that eventually must so level practice that the financial rewards will be universally unequal to holding the profession of dentistry to a social equality with other callings.

Dr. Crouse, at a meeting of the Chicago Club, said, in regard to this subject, "It is not a good paying business, and a man who has ability enough to do something else and knows it, won't go into it. I have not induced any one in the last twenty years to take it up as a life-time work."

Dr. A. W. Harlan added, "Probably there is not a man here who earns twenty-five thousand dollars a year, and I feel sure if I were practising as a lawyer that I would earn double that. . . . I do not believe there is a man in the room that ever did earn twenty-five thousand dollars a year, but I know of an eminent surgeon in this city who made seventy-five thousand dollars in one year. . . . There are too many mediocre intellects engaged in this occupation. And if men are content to live along on incomes of two thousand a year, or three thousand a year, and our profession is filled up with that kind of men, it is very hard for those who have high ideals to reach a high standard."

Dr. Allport followed with the statement,—"Dr. Harlan says there are very few of the dentists in our city doing a business of twenty-five thousand dollars per year, and many not over two thousand dollars. This estimate is too high, and I challenge any one to show that out of nearly seven hundred dentists in Chicago we have five who are doing a business of over fifteen thousand dollars per year, and that there are ten more who are doing a business of over ten thousand dollars, or twenty who are doing over five thousand dollars a year, while there are large numbers whose net income will not reach one thousand dollars."

These remarks from men of large experience in dental practice in a city supposed to yield the richest returns of any other outside, possibly, of New York, read strangely. To find these men sounding the note of alarm, and also placing themselves in the confessional and acknowledging that dentistry is a non-remunerative calling, is startling; that, notwithstanding the increased intelligence in a sanitary direction, the people will not pay the dentist as they pay, willingly or unwillingly, the doctor or lawyer.

While the main facts of these different statements are probably true, not only of Chicago but of the entire country, we might even go further and doubt whether any man ever succeeded in accumulating from his practice *alone* one hundred thousand dollars. This statement has, of course, no reliable data to sustain it; but experience leads to this conclusion, for of all the dentists who have left comparatively large estates, there have been other avenues for the wealth accumulated besides that of the daily work over the chair.

While it is true that the same amount of energy and active thought given to another line of work may show more brilliant results in individual cases, it does not by any means demonstrate, taking an equal number of men, or the entire body of dentists in the United States, and scattering them throughout all the avenues of active life, that the success would be any greater.

The wealth of the world has always been centred in the hands of the few, and, as was well remarked by Dr. Hartt in that same discussion, "We will have to go back farther than the dental colleges; . . . the poor man is getting poorer every day and the rich are getting richer." This is the rule of all centralization. It applies to power as well as money. Royalty and nobility mean the loss of freedom to the masses, and the accumulation of money in the hands of the few means, by the same law, poverty for the millions. It is not a law applicable to dentistry alone, but applies with equal force to the entire social fabric.

It is probably true that the emoluments of dental practice, outside of our large cities, will not average two thousand dollars a year, but those who have had experience in country practice will accord with the statement that but few in the ordinary branches of trade exceed this in the same locality. The laws of demand and supply apply equally to all classes and affect all alike.

The assertion that dentistry is becoming overcrowded is probably correct, and the usual charge is made that the colleges are exerting all the means in their power to draw young men into a study which can only end disastrously to them. While this may be also true of a few of these institutions, it is certainly not of those with which we are familiar, and we question the accuracy of the statement. As far as observation goes, the same effort is put forth that all medical colleges and institutions of learning make,—that of annual announcements and proper advertisements. Beyond this no well-regulated school would attempt to go.

The explanation of this rush for dentistry has a deeper explanation than that offered. It lies in the fact that large numbers of young men are reaching maturity in this country with no training beyond that the high school or college has afforded. With limited means they are called to meet face to face the struggle for bread. They may or may not be fitted by nature for commerce, or for occupations requiring mechanical tastes; this has yet to be tested in the marts and the shops of the world. The largest proportion look to the professions. The comparative advantages of medicine, law, theology, and dentistry are carefully weighed by parents and guardians, and a certain proportion go to each without much regard, it is feared, to fitness for either. The increasing number drawn to the latter is easily accounted for, from the fact that medicine and law are supposed to be overcrowded and slow to prove self-supporting.

Admitting all this, can that profession be considered non-remunerative which furnishes a good living to every man who will faithfully follow it? It is questionable whether more than this, or as much, can be said of any calling. Time alone will bring about a proper equilibrium between population and demand, but as yet the supply of young dentists hardly keeps pace with the growing needs, caused equally by enlargement of population as well as increased intelligence among the people.

The question may be asked, "What is success in life?" Is it to be counted at the close by the number of dollars stored away in investments? Is the record best made in the surrounding of one's self with luxuries? Is that life most to be commended that has been immured in an office, gathering the labors of other men for selfish purposes and giving nothing out? Is the largest bank account the best passport to a life eternal? These and many more questions of similar import might be propounded, and the right thinker would be forced to answer *no* to each.

The noble life is the one satisfied with a sufficiency to meet all ordinary wants. The true scientist, the teacher, the man who would rather build one stone upon another that the world may be a step higher when he leaves it, marks a success that the millionaires of the hour can never reach. Wealth melts before the rising sun of every recurring day and leaves no trace. The services in the sanctuaries of science mark epochs, and the impression is never effaced. Special arts may be lost, but the currents of civilization find their way always to new regions and new peoples. We build to-day upon the past, and upon its wealth of thought create new ideas, new combinations. This is true success, and finds its application in all the relations of life.

